# Identification of the interacting partners of a lysosomal membrane protein in living cells by BioID technique

**DOI:** 10.1016/j.xpro.2022.101263

**Published:** 2022-03-31

**Authors:** Dat Nguyen-Tien, Takehiro Suzuki, Toshihiko Kobayashi, Noriko Toyama-Sorimachi, Naoshi Dohmae

**Affiliations:** 1 Department of Molecular Immunology and Inflammation, Research Institute, National Center for Global Health and Medicine, 1-21-1 Toyama, Shinjuku, Tokyo 162-8655, Japan; 2 Biomolecular Characterization Unit, RIKEN Center for Sustainable Resource Science, Saitama 351-0198, Japan

**Keywords:** Cell-based Assays, Molecular Biology, Protein Biochemistry, Proteomics, Mass Spectrometry

## Abstract

The purpose of this protocol is to screen and identify the physiologically relevant interactors of a lysosomal protein in living cells. Here, we describe how to identify solute carrier family 15 member 4 (SLC15A4)-interacting proteins by BioID and mass spectrometry analysis. This protocol utilizes fusion of SLC15A4 with a mutant form of biotin ligase, BirA. The fusion protein can promiscuously biotinylate the proteins proximal to SLC15A4. The biotinylated endogenous proteins are pulled down by magnetic streptavidin beads and detected by mass spectrometry analysis.

For complete details on the use and execution of this protocol, please refer to Kobayashi et al. (2021).

## Highlights

• Exploring the relevant interactors of a lysosomal protein in its native environment
• Biotinylation and pulldown method of the proximal proteins with BioID fusion protein
• Detailed steps of sample preparation for mass spectrometer and quantification analysis

## Graphical abstract

**Figure:**
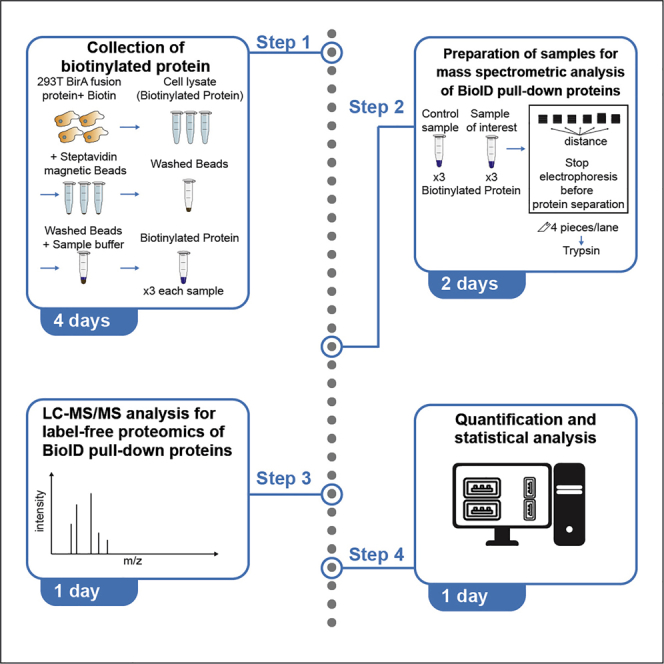

## Before you begin

This protocol was used to identify the interacting proteins of solute carrier family 15 member 4 (SLC15A4), a lysosome-resident amino acid/oligopeptide transporter, by BioID and mass spectrometry analysis (Kobayashi et al., 2021). The co-immunoprecipitation (Co-IP) is a conventional technique to identify protein-protein interactions. However, it has limitations in membrane proteins like SLC15A4 because of protein solubility or aggregation/filament formation in Co-IP lysis buffer. BioID, a new method to screen for physiologically relevant protein interactions based on a promiscuous biotin ligase (BirA) in a natural cellular setting (Roux et al., 2013), has advantages to overcome the Co-IP limitations because all of the cellular proteins are denatured and solubilized, and only those that are labeled with biotin are selectively captured. Here, we generated Control-BirA or BirA-SLC15A4-expressing HEK293T cells. The fusion ligases biotinylate adjacent endogenous proteins, which can be pulled down by magnetic streptavidin beads. These biotinylated proteins were identified by mass spectrometry. After identifying biotinylated proteins in each sample, we compared the proteins detected in the control samples (BirA only) with the BirA-SLC15A4 samples. The proteins enriched in the BirA-SLC15A4 sample represent the potentially SLC15A4-interacting candidates.

### Generation of cells expressing a BirA fusion protein

**Timing: 4 weeks**

As an example, we constructed the cMyc-BirA-SLC15A4 (hereafter BirA-SLC15A4) expression vector by subcloning full-length human SLC15A4 cDNA into the pcDNA3.1 mycBioID vector (Addgene) and further subcloned into pEBMulti-Bsd (FUJIFILM Wako Chemicals) to obtain the blasticidin S-resistance. BirA protein was fused at the N-terminal of SLC15A4. BirA-SLC15A4 expression vector was transfected into HEK293T cells using Lipofectamine 3000 Transfection Reagent (Thermo Fisher Scientific) to generate stably expressing cells; stable transfectants were selected in 10 μg/mL blasticidin S (FUJIFILM Wako Chemicals). We created a control cell line by transfecting HEK293T cells with a pEBMulti-Met-Gly-Cys-cMyc-BirA vector (Cont-BirA). The MGC motif helps to anchor cMyc-BirA to the cell membrane, which is related subcellular localization with BirA-SLC15A4.

### Confirm the expression of BirA fusion protein

**Timing: 3–4 days**

We performed flow cytometric analysis with c-Myc staining to confirm the expression of BioID constructs and western blotting with streptavidin-HRP (Thermo Fisher Scientific) to confirm the activity of BioID before scaling up for BioID pull down assays. If necessary, single cell cloning by limited dilution should be performed to choose the cell lines with comparable expression level. If necessary, cell viability and/or cytotoxicity assays should be performed because some recombinant fusion proteins have cytotoxic effects.

In addition, it is important to confirm the subcellular localization of the BirA fusion protein using fluorescence imaging whether it shows the same localization as the endogenous protein. In our case, we confirmed that both endogenous SLC15A4 and BirA-SLC15A4 showed the localization in LAMP1^+^ compartment.

## Key resources table

**Table:** 

REAGENT or RESOURCE	SOURCE	IDENTIFIER
**Antibodies**		

c-Myc Monoclonal Antibody (9E10), Alexa Fluor 555 (dilute 1:100)	Thermo Fisher Scientific	Cat# MA1-980-A555
Pierce™ High Sensitivity Streptavidin-HRP (dilute 1:10000)	Thermo Fisher Scientific	Cat# 21130
PE Rat anti-Mouse CD107a (LAMP1) (dilute 1:100)	BD Biosciences	Cat# 558661

**Chemicals, peptides, and recombinant proteins**		

Blasticidin S	Nacalai Tesque	Cat# ant-bl-05
(+)-Biotin	FUJIFILM Wako Chemicals	Cat# 023-08716
D-MEM (high-glucose)	Nacalai Tesque	Cat# 08458-16
Fetal Bovine Serum (FBS)	Biowest	Cat# S1820-500
Penicillin-Streptomycin Mixed Solution (PC/SM)	Nacalai Tesque	Cat# 09367-34
Lipofectamine™ 3000 Transfection Reagent	Thermo Fisher Scientific	Cat# L3000001
0.22-μM syringe-driven filter unit	Merck Millipore	Cat# SLGVR33RS
NaCl	Nacalai Tesque	Cat# 31320-05
Sodium Lauryl Sulfate (SDS)	Nacalai Tesque	Cat# 30400-85
Halt™ Protease Inhibitor Cocktail (100×)	Thermo Fisher Scientific	Cat# 78429
DC protein assay kit	Bio-Rad Laboratories	Cat# 5000111JA
Bovine Serum Albumin (BSA) Standard Ampules, 2 mg/mL	Thermo Fisher Scientific	Cat# 23209
Sodium deoxycholate	FUJIFILM Wako Chemicals	Cat# 192-08312
Triton X-100	Sigma-Aldrich	Cat# T9284
Dynabeads MyOne Streptavidin C1 (magnetic streptavidin beads)	Thermo Fisher Scientific	Cat# 65002
HEPES	Nacalai Tesque	Cat# 17514-15
NP-40 (Nonidet P40)	Nacalai Tesque	Cat# 25223-04
LiCl	Nacalai Tesque	Cat# 20624-62
Sample Buffer, Laemmli 2× Concentrate	Sigma-Aldrich	Cat# S3401-10VL
Pierce Silver Stain for Mass Spectrometry	Thermo Fisher Scientific	Cat# 24600
Acetonitrile	Thermo Fisher Scientific	Cat# A995-212
Formic acid, 99.0+%, Optima LC/MS grade	Fisher Scientific	Cat# A117-50
n-Dodecyl-β-D-maltoside (DM)	Dojindo	Cat# D316
Citric Acid Monohydrate	Nacalai Tesque	Cat# 09106-02
CBB G-250	Nacalai Tesque	Cat# 094-09
Polyvinylpyrrolidone (PVP-40)	Sigma-Aldrich	Cat# PVP-40-500G
Trypsin (N-TosylL-phenylalanyl chloromethyl ketone treated	Worthington Biochemical Corporation	Cat# TRTPCK
Acrylamide	FUJIFILM Wako Chemicals	Cat# 011-08015
(+/-)-Dithiothreitol (DTT)	FUJIFILM Wako Chemicals	Cat# 048-29224
Methanol	Junsei Chemical, Co.	Cat# 73125-0330
Guanidine Hydrochloride	Kanto Chemical	Cat# 17051-08
EDTA・2NA	Dojindo	Cat# 343-01861
Trizma (R) base	Sigma-Aldrich	Cat# T1503

**Deposited data**		

Raw and analyzed data	ProteomeXchange Consortium	https://www.ebi.ac.uk/pride/archive/projects/PXD020370

**Experimental models: Cell lines**		

Cont-BirA HEK293T	Kobayashi et al., 2021	N/A
BirA-SLC15A4 HEK293T	Kobayashi et al., 2021	N/A

**Recombinant DNA**		

pcDNA3.1 mycBioID	Roux et al., 2012	Addgene plasmid # 35700
pEBMulti-Bsd	FUJIFILM Wako Chemicals	Cat# 055-08311

**Software and algorithms**		

Mascot Server 2.6	Matrix Science	https://www.matrixscience.com/
Proteome Discoverer 2.2	Thermo Fisher Scientific	https://www.thermofisher.com/order/catalog/product/OPTON-31040

**Other**		

Q Exactive HF-X Hybrid Quadrupole-Orbitrap Mass Spectrometer	Thermo Fisher Scientific	Cat# 0726042
Easy nLC-1200	Thermo Fisher Scientific	Cat# LC140
Nano spray column NTCC-360/75–3-105,	Nikkyo Technos, Co.	Cat# NTCC-360/75-3-105
SpeedVac vacuum concentrator	N/A	N/A
Magnetic Stand (for 1.5 mL tube)	Tamagawa Seiki Co.	Cat# TA4899N12
Ultrasonic Homogenizer UH-50	SMT Corp.	UH-50

## Materials and equipment

**Table:** Complete medium

Reagent	Final concentration	Amount
D-MEM	–	500
FBS	10%	50 mL
PC/SM	1%	5 mL
**Total**	**–**	**555 mL**

Store at 4°C.

**Table:** 1 mM (20×) biotin buffer

Reagent	Final concentration	Amount
Biotin	1 mM	12.2 mg
D-MEM	–	50 mL
**Total**	**–**	**50 mL**

Sterilize by passing through a 0.22-μM syringe-driven filter unit. Aliquot and store at −20°C.

**Table:** PBS/EDTA

Reagent	Final concentration	Amount
10× PBS	1×	50 mL
0.5 M EDTA pH 8	1.8% (54 mM)	54 mL
Ultra-pure water	–	Fill up to 500 mL
**Total**	**–**	**500 mL**

Sterilize by autoclave and store at 4°C.

**Table:** Cell-suspension buffer

Reagent	Final concentration	Amount
1 M Tris-HCl, pH 7.5	50 mM	250 μL
5 M NaCl	500 mM	500 μL
100× Protease inhibitor cocktail	1×	50 μL
0.5 M EDTA pH 8	5 mM	50 μL
Ultra-pure water	–	Fill up to 5 mL
**Total**	**–**	**5 mL**

Use freshly prepared solution.

**Table:** Lysis buffer

Reagent	Final concentration	Amount
1 M Tris-HCl, pH7.5	50 mM	0.25 mL
5 M NaCl	500 mM	0.5 mL
2% SDS (w/v)	0.4% (w/v)	1 mL
100× Protease inhibitor cocktail	1×	50 μL
0.5 M EDTA pH 8	5 mM	50 μL
Ultra-pure water	–	Fill up to 5 mL
**Total**	**–**	**5 mL**

Use freshly prepared solution.

**Table:** Mix buffer

Reagent	Final concentration	Amount
Cell-suspension buffer	25% (v/v)	1.25 mL
Lysis buffer	25% (v/v)	1.25 mL
50 mM Tris-HCl, pH 7.5	50% (v/v)	2.5 mL
**Total**	**–**	**5 mL**

Use freshly prepared solution.

**Table:** Wash buffer 1

Reagent	Final concentration	Amount
SDS	2% (w/v)	0.5 g
Ultra-pure water	–	Fill up to 25 mL
**Total**	**–**	**25 mL**

Store up 2 weeks at room temperature (20°C–25°C); also use this buffer as a SDS stock solution for lysis buffer.

**Table:** Wash buffer 2

Reagent	Final concentration	Amount
10% Sodium deoxycholate (w/v)	0.1%	0.1 mL
20% Triton X-100	1%	0.5 mL
0.5 M EDTA pH 8	1 mM	20 μL
5 M NaCl	500 mM	1 mL
1 M HEPES pH 7.5	50 mM	0.5 mL
Ultra-pure water	–	Fill up to 10 mL
**Total**	**–**	**10 mL**

Store up to 2 weeks at room temperature (20°C–25°C).

Sodium deoxycholate stock solution must be protected from light.

**Table:** Wash buffer 3

Reagent	Final concentration	Amount
10% Sodium deoxycholate (w/v)	0.5%	0.5 mL
20% NP-40	0.5%	0.25 mL
0.5 M EDTA pH 8	1 mM	20 μL
2.5 M LiCl	500 mM	1 mL
1 M Tris-HCl, pH 7.5	10 mM	0.1 mL
Ultra-pure water	–	Fill up to 10 mL
**Total**	**–**	**10 mL**

Store up to 2 weeks at room temperature (20°C–25°C).

Sodium deoxycholate stock solution must be protected from light.

**Table:** CGP staining solution

Reagent	Final concentration	Amount
CBB G-250	0.05%	0.025 g
PVP-40	2%	1 g
Citric acid	20%	10 g
Ultra-pure water	–	Fill up to 50 mL
**Total**	**–**	**50 mL**

Use freshly prepared solution.

**Table:** De-staining buffer

Reagent	Final concentration	Amount
100% Acetonitrile	30%	3 mL
2 M Tris-HCl (pH 8.0)	10 mM	50 μL
Ultra-pure water	–	6.95 mL
**Total**	**–**	**10 mL**

Use freshly prepared buffer.

**Table:** Reducing buffer

Reagent	Final concentration	Amount
8 M guanidine-HCl / 1 M Tris-HCl (pH8.5) / 10 mM EDTA Stock solution	4 M guanidine-HCl / 0.5 M Tris-HCl (pH8.5) / 5 mM EDTA	0.5 mL
100 mM DTT	50 mM	0.5 mL
**Total**	**–**	**1 mL**

Use freshly prepared buffer.

**Table:** Alkylating buffer

Reagent	Final concentration	Amount
8 M guanidine-HCl / 1 M Tris-HCl (pH8.5) / 10 mM EDTA Stock solution	4 M guanidine-HCl / 0.5 M Tris-HCl (pH8.5) / 5 mM EDTA	0.5 mL
200 mM Acrylamide	100 mM	0.5 mL
**Total**	**–**	**1 mL**

Use freshly prepared buffer.

**Table:** Digestion buffer

Reagent	Final concentration	Amount
2 M Tris-HCl (pH 8.0)	20 mM	100 μL
0.5% DM	0.025%	50 μL
Ultra-pure water	**–**	0.985 mL
**Total**	**–**	**1 mL**

Can be stored for at least a month at 4°C.

## Step-by-step method details

### Collection of biotinylated proteins

**Timing: 4 days**

**Figure 1:**
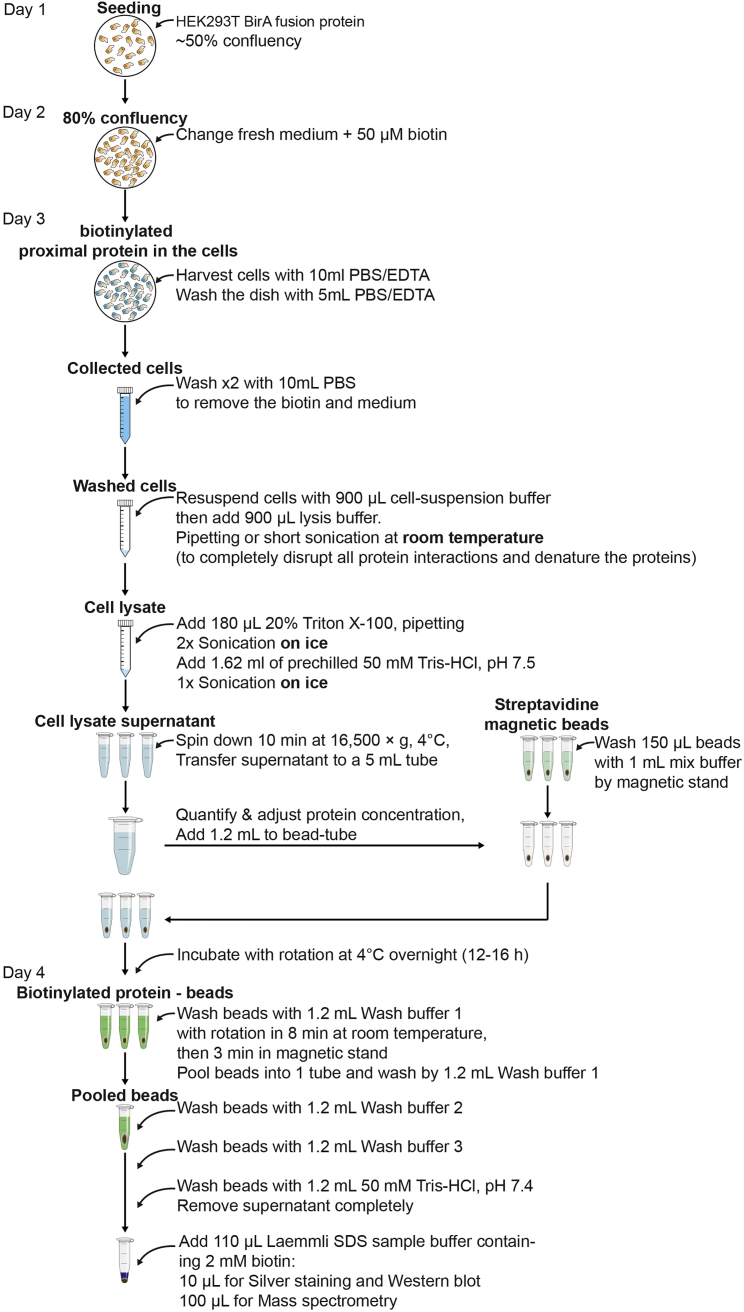
Outline of collecting biotinylated proteins The following step-by-step outline from BirA fusion protein expressing HEK293T cells to biotinylated precipitates.

Preparation of samples with sufficient amount of protein biotinylated by the BioID fusion protein is identified by mass spectrometry (Figure 1). At the starting point, the amount of total protein per sample is about 20 mg.

***Note:*** Prepare biological triplicate samples to see reproducibility in the next mass spectrometric analysis. All steps hereafter are carried out using DNase/RNase-free tubes that have not previously opened and a tube cap opener, and wearing gloves, face mask, safety goggle and head net, to avoid contamination of the samples, particularly with keratin. Should perform the experiment inside a clean bench.

#### Perform cell lysis

1. Begin with one 15-cm dish (or three 10-cm dishes) for each sample (HEK293T cells expressing BioID fusion protein or BioID constructs).
2. When cells reach 80% confluency, change medium to fresh complete medium containing 50 μM biotin (1×).
3. Incubate cells for 16 h–18 h.
4. Collect the cells.
  a. Remove medium completely by aspiration.
  b. Collect the cells by pipetting with 10 mL/dish of PBS/EDTA into 15 mL tube.
  c. Wash the dish one more time with 5 mL PBS/EDTA to collect all residue cells.
  d. Spin down for 5 min at 400 × g at room temperature (20°C–25°C).
5. Discard the supernatant and wash the cells twice by spin down for 5 min at 400 × g at room temperature (20°C–25°C) with 10 mL/tube of PBS to remove all residue biotin.
6. Lyse the cells. This step is to completely disrupt all protein interactions and denature proteins.
  a. Resuspend the cells in 900 μL cell-suspension buffer (the cells need to be completely suspended for sufficient lysis).
  b. Add 900 μL lysis buffer and pipette strongly the solution but avoid making the bubble. Perform this step at room temperature (20°C–25°C).

***Note:*** If it is hard to pipette the solution due to DNA genome releasing, apply a short sonication at 30% amplitude for 15–30 s at room temperature (20°C–25°C).

7. Add 180 μL of 20% Triton X-100 and mix by pipetting (to prevent SDS precipitation on ice). From now keep the tubes on ice for subsequent sonication.
8. Position the sonicator probe tip in the sample just above the tube bottom to avoid making the bubble.
9. Sonicate samples.
  a. Apply sonication for two sessions for 30 s using an ultrasonic homogenizer at 30% amplitude.
  b. Then let the tube rest on ice for 2 min between each session to prevent overheating.
  c. During the resting time, apply the sonication for other samples after washing the probe tip with 70% ethanol and ultra-pure water.
10. Add 1.62 mL of prechilled 50 mM Tris-HCl, pH 7.5, and mix well.
11. Repeat one more session of sonication as mentioned in steps 8 and 9.
12. Aliquot the sample evenly into three prechilled 1.5-mL tubes.

***Note:*** This step can be excluded if the rotor for 5 ml tube is available.

13. Spin down for 10 min at 16,500 × g, 4°C. Carefully transfer all supernatant of the same sample to a prechilled 5 mL tube.

***Note:*** Do not disturb the small insoluble pellet on the tube wall when transferring the supernatant.

14. Measure protein concentration by using the DC protein assay kit (microplate assay) with BSA as a standard. Adjust the protein concentration of samples to the lowest concentration samples with the mix buffer. Keep samples on ice until use.

#### Perform affinity purification of biotinylated proteins

(Steps 15–17 are to equilibrate the magnetic streptavidin beads with the mix buffer).

15. During steps 13 and 14, place three new 1.5-mL tubes in the Tamagawa magnetic stand (TA4899N12). Add 1 mL of the mix buffer to each tube.
16. Mix the stock of magnetic streptavidin beads well with gentle tapping to resuspend and add 150 μL of beads to each tube prepared in step 15. Wait for 3 min at room temperature (20°C–25°C).
17. Once the beads accumulate at one side of tube wall, remove the supernatant gently by 1-mL pipette.

(Continue with the samples from step 14).

18. Divide 1.2 mL of sample from step 14 to the three tubes containing equilibrated beads.
19. Resuspend the samples and beads with gentle pipetting.
20. Incubate the tube on a rotator at 4°C for overnight (at least 12 h).
21. Place the tubes on the magnetic stand and wait for 3 min to collect beads at room temperature (20°C–25°C).
22. Remove the supernatant gently by pipetting. Try not to disturb beads on the wall of the tube.
23. Add 1.2 mL of wash buffer 1 to each tube and resuspend beads gently by pipetting.
24. Rinse the beads with rotation in a rotator for 8 min at room temperature (20°C–25°C).
25. Remove the supernatant as described in steps 21 and 22.

(Pool beads from same samples into one tube).

26. Add 1.2 mL of wash buffer 1 to one of the three tubes and resuspend gently.
27. Transfer resuspended sample from step 26 to the second of the three tubes, and resuspend gently. Repeat this step to pool all beads from same samples into one tube in a final volume of 1.2 mL wash buffer 1.

**Figure 2:**
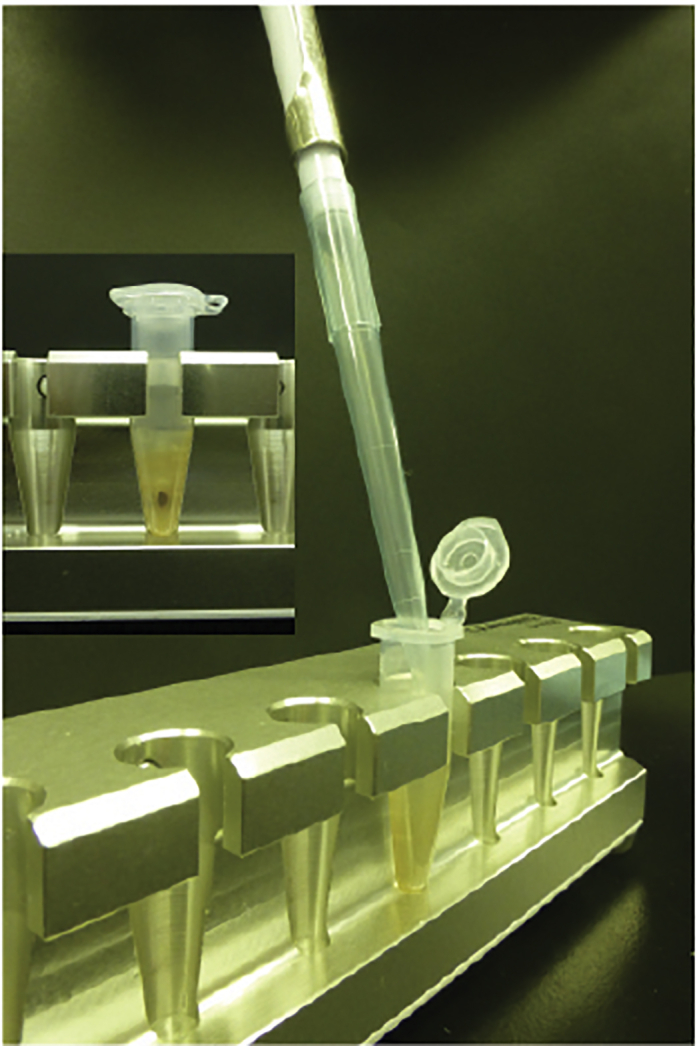
The tip to pipetting for beads washing steps (steps 22–31) The tip should be held at a 30°–40° angle and immersed about 1–2 mm. Move down the tip when aspirating the washing buffer.

***Note:*** From this step, it is easy to lose beads, be careful with pipetting (Figure 2).

**Figure 3:**
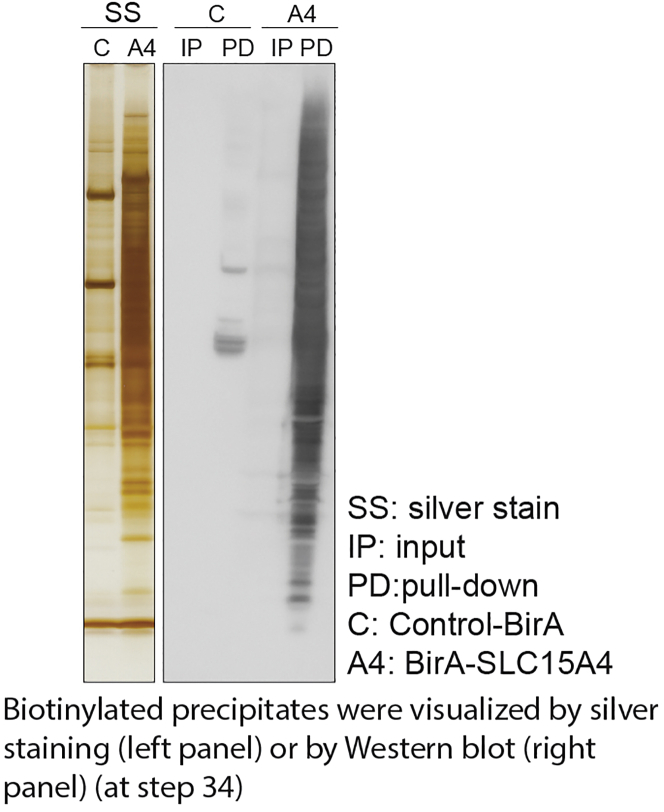
Precipitation of BirA fusion protein Control-BirA and BirA-SLC15A4 Biotinylated precipitates were visualized by silver staining (left panel) or by Western blot (right panel) (at step 34).

28. Place the combined tube on a rotator for 8 min at room temperature (20°C–25°C).
29. Wash once with 1.2 mL of wash buffer 2 as described in steps 21 through 25.
30. Wash once with 1.2 mL of wash buffer 3 as described in steps 21 through 25.
31. Wash once with 1.2 mL of 50 mM Tris-HCl, pH 7.4 as described in steps 21 through 25. (troubleshooting 1).
32. Remove the supernatant completely. Do not disrupt beads at the tube wall.
33. Add 110 μL Laemmli SDS sample buffer containing 2 mM biotin.
34. Save 10 μL (10% of total) of sample for further analysis by silver stain or western blot (Figure 3). 100 μL of sample will be used for mass spectrometric analysis.

**Pause Point:** If necessary, freeze the sample quickly in liquid nitrogen. Frozen samples can be stored at −80°C until performing mass spectrometric analysis.

### Preparation of samples for mass spectrometric analysis of BioID pull-down proteins

**Timing: 2 days**

**Figure 4:**
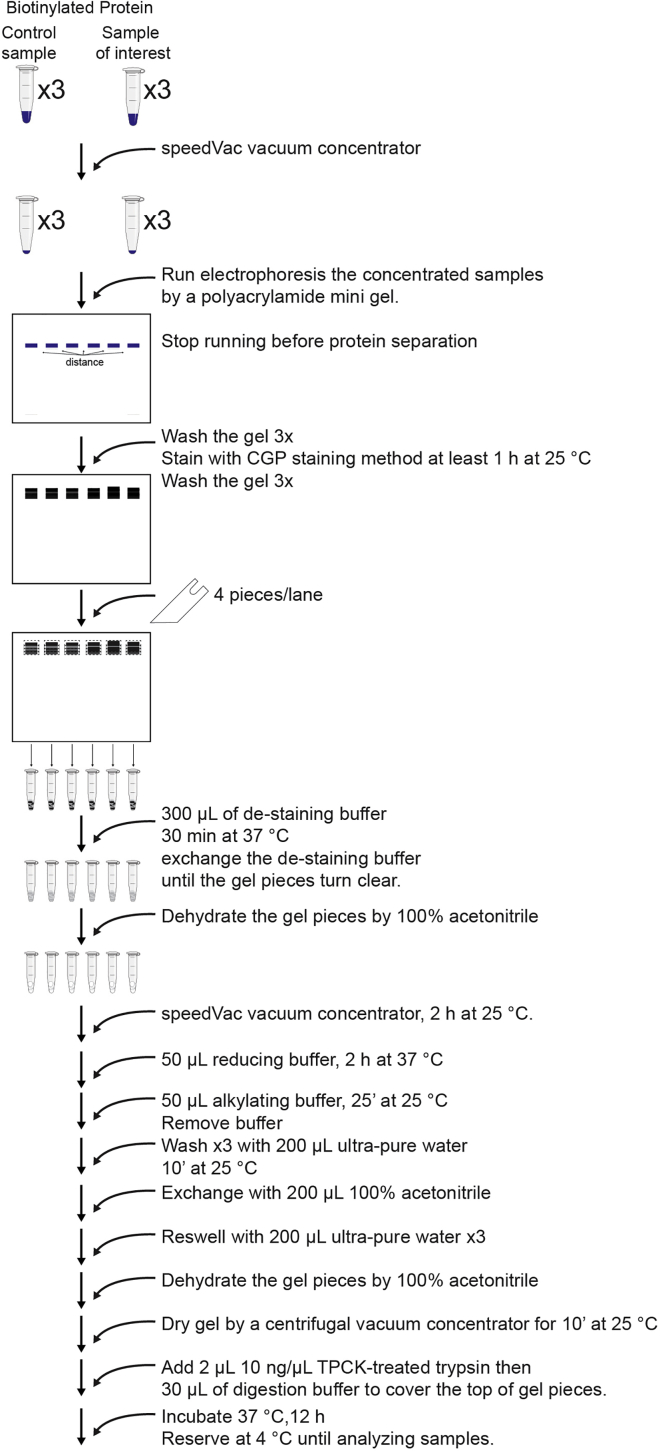
Outline of mass spectrometric sample preparation The following step-by-step outline from biotinylated precipitates to mass-spec-ready samples.

Single-band focused SDS-PAGE containing whole proteins and its in-gel digestion with trypsin (Figure 4).

***Note:*** Prepare biological triplicate samples to see reproducibility in the next mass spectrometric analysis. To avoid modification by unreacted acrylamide, the one-day-old gel is used. All steps after are carried out not to contaminate the samples with keratin.

35. Elute the streptavidin bound proteins with 100 μL Laemmli SDS sample buffer containing 2 mM biotin at 98°C.
36. Concentrate the samples to the volume of 20 μL with a SpeedVac vacuum concentrator for 2 h at 25°C.
37. Load the concentrated samples onto a polyacrylamide mini gel.

***Note:*** If there are multiple samples, load each sample onto a gel at a distance to avoid cross-contamination.

38. Stop the electrophoresis when the dye front migrated from the stacking gel to the separation gel.
39. Wash the gel three times with 200 mL of ultra-pure water using a shaker and discard the washing solution.
40. Stain the gel according to the CGP stain method (Yasumitsu et al., 2010). Pour 50 mL of the CGP staining solution into the gel, and incubate for at least 1 h at 25°C.
41. Discard the staining solution and wash the gel three times with 200 mL ultra-pure water for 1 h to visualize the protein bands. In each lane, a single band is observed as a mixture of all the proteins.
42. Cut the visualized band into the four pieces using a clean razor blade and transfer them to a 600 μL microcentrifuge tube.
43. Soak the gel pieces in 300 μL of de-staining buffer to de-stain CBB G-250. Incubate for 30 min at 37°C and exchange the de-staining buffer until the gel pieces turn clear.
44. Dehydrate the gel pieces by adding 100% acetonitrile. Exchange 100% acetonitrile until the gel pieces turn white. Discard the acetonitrile and dry the gel pieces completely with a SpeedVac vacuum concentrator for 10 min at 25°C.
45. Add 50 μL of reducing buffer onto the dried gel pieces and incubate for 2 h at 37°C.
46. Add 50 μL of alkylating buffer onto the reduced gel pieces and incubate for 30 min at 25°C. Remove the buffer after alkylation by pipetting.
47. Wash gel pieces three times with 200 μL of ultra-pure water for 10 min at 25°C. Exchange washing solution with 200 μL of 100% acetonitrile and reswell with 200 μL of ultra-pure water. This step is repeated three times.

***Note:*** It is advisable to wash over three times to remove any reagents that may affect the subsequent mass spectrometric analysis.

48. Remove the washing solution and dehydrate the gel pieces with 100% acetonitrile, Dry the gel pieces with a centrifugal vacuum concentrator for 10 min at 25°C.
49. Reswell the dried gels completely with 2 μL of 10 ng/μL TPCK-treated trypsin. Add 30 μL of digestion buffer to cover the top of gel pieces.

***Note:*** The standard protocol is for about 1 μg protein, and the amount of enzyme and buffer are changed according to the amount of protein.

50. Incubate samples for 12 h at 37°C to digest with trypsin. The resulting peptides can be stored at 4°C for 1 week.

**Pause Point:** At this time, the peptide mixture can be safely stored at −20°C for one month.

### LC-MS/MS analysis for label-free proteomics of BioID pull-down proteins

**Timing: 1 day**

Peptide mixture generated by carrying out steps 35–50 is analyzed using LC-MS/MS. The obtained data are used for the next label-free quantitative analysis.

***Note:*** Prepare biological triplicate samples to see reproducibility and all samples are analyzed in the same volume for use in the next step of comparative quantification.

51. Transfer the digested peptides mixture to the vials in the LC autosampler and acidify the peptides solution by adding 1% formic acid. Adjust all sample volumes to equal. (troubleshooting 2).
52. Analyze the digestion mixtures with a nanospray column (NTCC-360/75–3-105, Nikkyo Technos, Co., Ltd.) using EASY-nLC 1200 (Thermo Fisher Scientific) coupled with a Q Exactive HF-X mass spectrometer (Thermo Fisher Scientific). LC-MS/MS data of three independent biological replicates are available through the ProteomeXchange: PXD020370) (troubleshooting 3).
  a. A flow rate of 300 nL/min was used and peptides were separated using a 50 min linear gradient of 0%–64% over B for 50 min (solvent A: 0.1% formic acid in water, solvent B: 0.1% formic acid in 80% acetonitrile).
  b. Carry out LC-MS/MS analysis according to the following parameters in Table 1. LC-MS/MS parameters are listed in Table 1.

**Table 1:** LC-MS/MS parameters for a label-free proteomics

Instrument	Q exactive HF-X
Spray Voltage	2.00 kV
Capillary Temp	250°C

**Full MS**	

Resolution	60,000
AGC target	3e6
Maximum IT	60 ms
Number of scan ranges	1
Scan range	200–2,000 m/z
Spectrum data type	Profile

**dd-MS2 /dd-SIM**	

Microscans	1
Resolution	15,000
AGC target	1e5
Maximum IT	85 ms
Loop count	10
MSX count	1
TopN	10
Isolation window	1.6 m/z
NCE/stepped NCE	27
Spectrum data type	Centroid

**dd Settings**	

Minimum AGC target	8.30e3
Intensity threshold	9.8e4
Charge exclusion	unassigned, 5–8, >8
Peptide match	Preferred
Exclude isotopes On Dynamic exclusion	20.0 s

All other settings use the default settings.

## Expected outcomes

**Figure 5:**
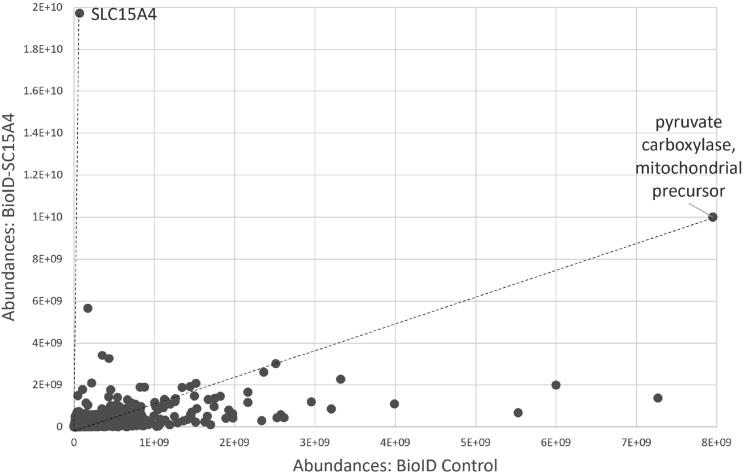
Intensity correlation plot between BioID experimental samples and control samples The ion intensity of proteins identified by BioID experiments is plotted. The dashed line connects each of the endogenous PC and SLC15A4 intensities to the zero point.

To check our protocol works well, an intensity correlation between BioID experimental samples and control samples was plotted in Figure 5. Pyruvate carboxylase (PC), an endogenous biotin-binding protein, and SLC15A4, a BirA fusion protein, are shown on the plot. SLC15A4 was detected with the highest intensity only in the BioID experimental sample, while PC was strongly detected in both BioID experimental samples and control samples. The results showed that BirA-SLC15A4 was specifically and strongly biotinylated by BioID experiments. Also, proteins with a higher abundance ratio than the endogenous PC are likely to have been specifically biotinylated by BioID experiments. Therefore, the proteins in the region between the dashed lines were treated as candidates for biotinylated proteins in the BioID experiment. As a result, 629 proteins could be identified as biotinylated proteins by BioID experiments. Furthermore, as indicated in the section on quantitative and statistical analysis and troubleshooting, it is possible to verify the validity of the results by creating several constructs and conducting the same experiment. Among the identified proteins, we could confirm the interaction between SLC15A4 and the lysosomal proteins involved in mTOR pathway by Co-IP assay (Kobayashi et al. 2021).

## Quantification and statistical analysis

**Timing: 1 day**

The LC-MS/MS data is processed by label-free proteomic analysis and the list of proteins that have been specifically increased by the BioID experiment is obtained. (troubleshooting 4, 5, 6).

***Note:*** The use of data from biological triplicate samples increases the reliability of the results.

1. Analyze LC-MS/MS data using Proteome Discoverer 2.2 (Thermo Scientific) combined with Mascot 2.6 (Matrix Science) for protein identification and label-free quantification.

***Note:*** The analysis software should be used Proteome Discoverer version 2.2 or higher. Also, Other MS analysis software like SEQUEST (Yates et al. 1995) and MaxQuant (Tyanova et al., 2016) can be used.

2. Read the resulting raw data of LC-MS/MS by Proteome Discoverer and set the names in “Study Factors” for each condition. e.g., ‘‘Control 1’’, ‘‘BioID sample 1’’.
3. Set up a “processing workflow” and a “consensus workflow”. Select the method for label-free quantification in the “processing workflow” of the Proteome Discoverer.
  a. Select “PWF_QE_Precursor_Quan_and_LFQ_SequestHT_Percolator” in the “ProcessingWF_QExactive” folder, which is the default workflow, and set Mascot node with the following parameters instead of Sequest. Database: NCBI-nr protein database (20160711, Taxonomy: H. sapiens 326,427 sequences), Parameters: enzyme = trypsin; maximum missed cleavages = 3; Fixed modifications = Propionamide (C); variable modifications = Acetyl (Protein N-term), Gln- > pyro-Glu (N-term Q), Oxidation (M); product mass tolerance = ± 15 ppm; product mass tolerance = ± 30 milli mass unit; instrument type = ESI-TRAP.
  b. Select “CWF_Comprehensive_Enhanced Annotation_LFQ_and_Precursor_Quan” in the “ConsensusWF” folder, which is the default consensus workflow. Use the default settings.
4. In the “Grouping & Quantification” section, set the protein abundance ratio. e.g., “BioID sample 1 / control 1”.
5. Run the program when the various settings are finished.

***Note:*** Make sure hard disk space is enough.

6. Once the analysis is complete, you can also view the results in Proteome Discoverer. It is also possible to output the data in Excel or other formats. Statistical analysis like PCA plot can be performed on Proteome Discoverer.
7. In this setting, proteins with a high protein abundance ratio can be considered specifically enriched by the BioID experiment. We set the threshold for the protein abundance ratio (BioID-SLC15A4 / BioID-control) below the value of endogenous PC, resulting in 629 proteins as candidates. As shown in a Note, the results were validated by focusing on those that showed a common increase between several experiments.

***Note:*** It is advisable to perform BioID experiments on other proteins like the target protein, or constructs with BirA fused to the N- and C-termini respectively. Proteins that are commonly increased in several experiments can be considered as interacting candidates, which helps to validate the results.

## Limitations

Limitations of BioID include the expression of an exogenous fusion protein. The addition of biotin ligase may impair normal targeting, stability, or function. Another limitation is that the identity of a candidate protein does not immediately imply an interaction with the bait. Biotinylated proteins resulting from the use of BioID fall under three categories: (i) direct interactions, (ii) indirect interactions, or (iii) proximal proteins that do not interact directly or indirectly. It is possible that false negatives may occur if candidates lack accessible primary amines for biotinylation. The permanent addition of biotin to primary amines may alter protein function by modifying charge and removing accessibility of those sites for alternative modifications. Negative consequences from expression of a BioID fusion protein in our case have not been observed, but must be considered possible.

## Troubleshooting

### Problem 1

Lose the bead in the washing steps (step-by-step method details steps 22–31).

### Potential solution

Pipetting the wash buffer containing the beads to a new tube, re-collect by magnetic stand and pool together with its sample in the next step.

### Problem 2

Difficult to de-stain CGP stain (step-by-step method details step 51).

### Potential solution

Proceed to the alkylation step and then carry out the de-staining step.

### Problem 3

Low detection sensitivity of peptides in LC-MS (step-by-step method details step 52).

### Potential solution

If the protein amounts are too low, start by re-preparing BioID samples to increase amounts of samples.

### Potential solution

Confirmation of in-gel digestion steps by peptide mass fingerprinting using MALDI-TOF MS.

### Potential solution

If it is an LC-MS problem, use a standard sample (e.g., tryptic Hela cell lysate) to check the condition of the instrument. For example, we analyzed 0.5 μg of a proteome sample of HL60 cells using our instruments and settings. As a result, 940 proteins and 2,967 peptides were identified with high confidence.

### Problem 4

Problems of quantification accuracy due to the accuracy of gel excision (quantification and statistical analysis).

### Potential solution

If further accuracy is required, digestion in a solution system should be considered.

### Problem 5

Differences in the efficiency of in-gel digestion between samples (quantification and statistical analysis).

### Potential solution

Examine the number of missed cleavages per sample and monitor the variability.

### Problem 6

Many false-positive candidates (quantification and statistical analysis).

### Potential solution

Perform BioID experiments with other proteins like the target protein, or proteins with BirA fused to the N- and C-termini respectively. The candidate proteins can be validated by comparing proteins that show similar behavior in these multiple experiments.

## Resource availability

### Lead contact

Further information and requests for resources and reagents should be directed to and will be fulfilled by the lead contact, Noriko Toyama-Sorimachi (nsorimachi@ri.ncgm.go.jp).

### Materials availability

All reagents generated in this study are available from the lead contacts upon completing a Materials Transfer Agreement.

## Data Availability

LC-MS/MS raw files and Proteome Discoverer output files are available at ProteomeXchange: PXD020370.
